# Genetic Ablation of Calcium-independent Phospholipase A_2_γ Exacerbates Glomerular Injury in Adriamycin Nephrosis in Mice

**DOI:** 10.1038/s41598-019-52834-x

**Published:** 2019-11-07

**Authors:** Hanan Elimam, Joan Papillon, Julie Guillemette, José R. Navarro-Betancourt, Andrey V. Cybulsky

**Affiliations:** 1 Department of Medicine, McGill University Health Centre Research Institute, McGill University, Montreal, Quebec Canada; 2 Department of Biochemistry, Faculty of Pharmacy, University of Sadat City, Monufia, Egypt

**Keywords:** Glomerular diseases, Kidney diseases

## Abstract

Genetic ablation of calcium-independent phospholipase A_2_γ (iPLA_2_γ) in mice results in marked damage of mitochondria and enhanced autophagy in glomerular visceral epithelial cells (GECs) or podocytes. The present study addresses the role of iPLA_2_γ in glomerular injury. In adriamycin nephrosis, deletion of iPLA_2_γ exacerbated albuminuria and reduced podocyte number. Glomerular LC3-II increased and p62 decreased in adriamycin-treated iPLA_2_γ knockout (KO) mice, compared with treated control, in keeping with increased autophagy in KO. iPLA_2_γ KO GECs in culture also demonstrated increased autophagy, compared with control GECs. iPLA_2_γ KO GECs showed a reduced oxygen consumption rate and increased phosphorylation of AMP kinase (pAMPK), consistent with mitochondrial dysfunction. Adriamycin further stimulated pAMPK and autophagy. After co-transfection of GECs with mito-YFP (to label mitochondria) and RFP-LC3 (to label autophagosomes), or RFP-LAMP1 (to label lysosomes), there was greater colocalization of mito-YFP with RFP-LC3-II and with RFP-LAMP1 in iPLA_2_γ KO GECs, compared with WT, indicating enhanced mitophagy in KO. Adriamycin increased mitophagy in WT cells. Thus, iPLA_2_γ has a cytoprotective function in the normal glomerulus and in glomerulopathy, as deletion of iPLA_2_γ leads to mitochondrial damage and impaired energy homeostasis, as well as autophagy and mitophagy.

## Introduction

Glomerular visceral epithelial cells (GECs) or podocytes play a critical role in the maintenance of glomerular permselectivity^1,2^. These cells have a complex morphology characterized by cell bodies with projecting interdigitating foot processes that are bridged by filtration slit diaphragms. The actin cytoskeleton provides support for their intricate shape. Podocytes are metabolically robust cells with high energy demands – they produce slit-diaphragm proteins, adhesion molecules and glomerular basement membrane (GBM) components. Podocyte injury, manifesting as proteinuria, is implicated in a number of glomerular diseases^1,2^. We reported previously that calcium-independent phospholipase A_2_γ (iPLA_2_γ) mRNA and protein are expressed in the glomerulus *in vivo*^3^. iPLA_2_γ is cytoprotective in complement-mediated GEC injury^3^. Moreover, genetic ablation of iPLA_2_γ in mice results in striking mitochondrial ultrastructural abnormalities and enhances the number of autophagosomes in podocytes, and leads to loss of podocytes in aging mice, without detectable albuminuria^4^. In anti-GBM nephritis, deletion of iPLA_2_γ exacerbated albuminuria. Thus, iPLA_2_γ has a protective functional role in the normal glomerulus and in glomerulonephritis. Our studies in cultured GECs verified that deletion of iPLA_2_γ is associated with mitochondrial dysfunction and enhanced autophagy^4^.

We and others have demonstrated that iPLA_2_γ is localized subcellularly at the endoplasmic reticulum and mitochondria, and localization is dependent on the N-terminal region of iPLA_2_γ^5–7^. iPLA_2_γ may be active under basal and stimulated conditions; the latter was dependent on phosphorylation at Ser-511 and/or Ser-515 via mitogen-activated protein kinase-interacting kinase 1 (MNK1)^6^. At the ER, iPLA_2_γ can modulate the unfolded protein response^8^. Phospholipases at the mitochondria have a crucial role in the regulation of mitochondrial function and signaling^5,9^. The role of iPLA_2_γ in mitochondrial bioenergetic function and its importance in cellular energy metabolism and homeostasis was previously identified in several tissues, including heart, skeletal muscle, liver, and brain^10–13^. iPLA_2_γ knockout (KO) mice display reduced growth rate, cold intolerance, and various bioenergetic dysfunctional phenotypes^10^. For example, iPLA_2_γ deletion induced marked disruption in mitochondrial phospholipid homeostasis in the brains of aging mice, resulting in enlarged and degenerating mitochondria, leading to enhanced autophagy and cognitive dysfunction^11^. A 7-year old human female with compound heterozygous mutations in the gene encoding iPLA_2_γ displayed a mitochondrial myopathy with dystonia, abnormal gait, seizures and lactic acidosis^14^. Thus, iPLA_2_γ plays an important role in mitochondrial lipid metabolism and membrane structure, and perturbation of this role affects fatty acid β-oxidation, oxygen consumption, energy expenditure, and tissue homeostasis.

Autophagy is an essential “self-eating” process that begins with formation of a double-membrane structure, the phagophore, which engulfs a portion of the cytoplasm^15–18^. Numerous proteins are involved in assembly of autophagosomes. Among these, microtubule-associated protein 1 light chain 3 (LC3-I) becomes lipidated (i.e. converted to LC3-II) and redistributes to autophagic vesicles. LC3-II is, therefore, commonly used as a marker of autophagy. Autophagosomes fuse with lysosomes to form autolysosomes. During this process, the contents of the autophagosomes, such as malformed proteins or damaged organelles, are degraded by lysosomal hydrolases. Autophagy recovers amino acids and fatty acids, thereby facilitating cell survival. Autophagy may be generalized (“macroautophagy”; here referred to as autophagy) or selective, such as mitophagy, where there is degradation of mitochondria by autophagy^17,19,20^. Proper mitochondrial quality and quantity are essential for normal cellular functions. Thus, a viable pool of mitochondria is maintained by continuous cycles of fusion and fission, as well as biogenesis and degradation, which produces new mitochondria and removes defective organelles. In fact, mitophagy (both receptor and nonreceptor-mediated mitophagy) is the principal mechanism for removing damaged or superfluous mitochondria, and it attenuates the potentially deleterious impact on cellular metabolism of damaged mitochondria^19,21^. It should be noted that disruption of autophagy in podocytes in mice leads to injury of these cells as mice age, implying that autophagy is an essential process for the maintenance of homeostasis in podocytes^16,18^.

In the present study, we further addressed the role of iPLA_2_γ in glomerular injury. Given the importance of mitochondrial function and autophagy in the maintenance of homeostasis in podocytes^16,22^, our focus has been on the interaction of iPLA_2_γ with these two processes. We demonstrate that deletion of iPLA_2_γ in young mice exacerbates podocyte injury and enhances autophagy in adriamycin nephrosis, an experimental model of human focal segmental glomerulosclerosis (FSGS). Moreover, in GECs derived from iPLA_2_γ KO mice, we show mitochondrial dysfunction, as well as enhanced autophagy and mitophagy.

## Results

### Deletion of iPLA_2_γ exacerbates albuminuria and reduces podocyte number in adriamycin nephrosis

**Figure 1:**
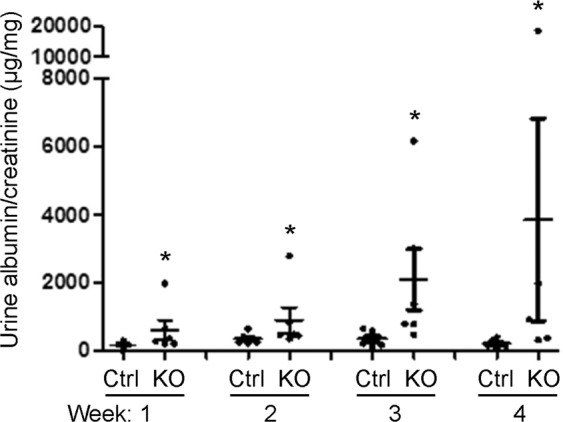
Deletion of iPLA_2_γ exacerbates albuminuria in adriamycin nephrosis. Control (Ctrl) and iPLA_2_γ KO mice were injected with adriamycin (12 mg/kg). Urine was collected at weekly intervals for up to 4 weeks. *P < 0.001 KO vs control; 6 mice per group.

Earlier, we demonstrated that podocytes in aging iPLA_2_γ KO mice (10–11 months of age) show injury to plasma membranes and mitochondria, as well as an increased number of autophagosomes, although these mice did not develop albuminuria^4^. In younger mice (~3 months of age), deletion of iPLA_2_γ exacerbated podocyte injury and albuminuria in acute anti-GBM nephritis^4^. In the present study, we addressed the functional role of iPLA_2_γ in injured podocytes by inducing adriamycin nephrosis in mice, a model of chronic proteinuric glomerular disease^23^. Adriamycin (doxorubicin) is believed to primarily target podocytes, and the proposed mechanisms of adriamycin-induced tissue damage are multiple, and may include reduction in mitochondrial DNA, introduction of DNA breaks, lipid peroxidation, protease inhibition, and disruption of the cytoskeleton^24–27^. Control and iPLA_2_γ KO mice (age 3.5–4.5 months) were injected with adriamycin, and urine was collected at weekly intervals for up to 4 weeks. At each time point, iPLA_2_γ KO mice showed greater albuminuria, compared with control (Fig. 1 and Supplementary Fig. 1). The control and KO groups each included 3 male and 3 female mice – 5 wild type (WT) and 1 iPLA_2_γ heterozygous mouse; since there were no apparent differences in albumin/creatinine levels between males and females, or WT and heterozygous, these results are presented together. The urine albumin/creatinine levels in adriamycin-treated control mice were not significantly different from untreated WT or KO mice reported earlier^4^, indicating that control mice were adriamycin-resistant at the dose of drug employed, while deletion of iPLA_2_γ sensitized mice to adriamycin-induced glomerular injury.

**Figure 2:**
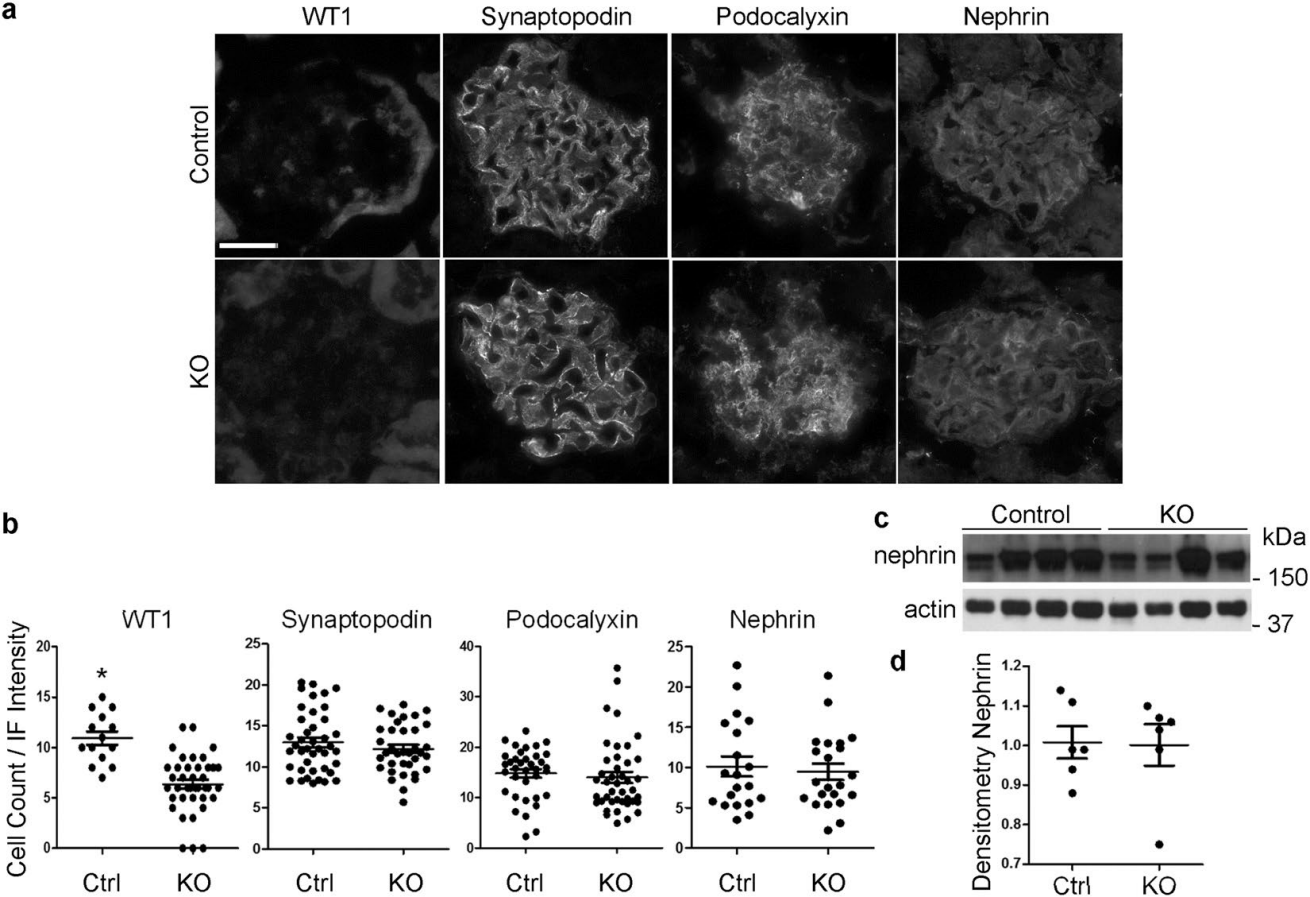
Effect of iPLA_2_γ on podocyte number and differentiation in adriamycin nephrosis. Kidneys were harvested from control (Ctrl) and iPLA_2_γ KO mice 4 weeks after adriamycin administration. (**a**,**b**) Kidney sections were stained with antibodies to WT1, synaptopodin, podocalyxin and nephrin. (**a**) Representative IF staining. (**b**) WT1 counts and quantification of IF intensity. The number of WT1 positive nuclei (reflecting number of podocytes) was lower in KO mice. *P < 0.0001 KO vs control, 14 measurements in control group (4 mice) and 39 in KO group (5 mice). There are no significant differences between control and KO mice in IF staining intensity of synaptopodin (41 measurements in control group, 6 mice, and 36 in KO group, 6 mice), podocalyxin (36 measurements in control group, 6 mice, and 42 in KO group, 6 mice), and nephrin (20 measurements in control group, 4 mice, and 22 in KO group, 4 mice). Bar = 25 µm. (**c**,**d**) Glomeruli were isolated from mouse kidneys, and lysates were immunoblotted with anti-nephrin antibody. (**c**) Immunoblot. An uncropped immunoblot is presented in Supplementary Fig. 5. (**d**) Densitometric quantification. There are no significant differences in nephrin expression between control and KO (6 mice per group).

To examine podocyte number and expression of podocyte differentiation parameters in adriamycin nephrosis, kidneys were harvested from control and iPLA_2_γ KO mice 4 weeks after adriamycin administration. The number of Wilm’s tumor-1 (WT1) positive nuclei (reflecting the number of podocytes) was ~40% lower in KO mice, compared with control (Fig. 2a,b), and there was no significant effect of adriamycin on cross-sectional glomerular area (control: 3198 ± 138 µm^2^ vs KO: 3046 ± 129 µm^2^; 37–43 glomeruli in 5–6 mice per group). The measurements of WT1 positive nuclei and glomerular area in the adriamycin-treated control mice in these experiments were very similar to basal values in WT mice either 3–4 or 10–11 months of age, reported earlier^4^. Thus, adriamycin induced podocyte depletion only in KO mice, and did not affect podocyte numbers in control. There were no significant differences between control and KO mice in the immunofluorescence (IF) staining pattern or intensity of synaptopodin, podocalyxin, and nephrin (Fig. 2a,b). Moreover, immunoblotting of lysates of isolated glomeruli (at 4 weeks) did not reveal significant differences in nephrin expression between control and KO mice (Fig. 2c,d). These results suggest that remaining injured podocytes may have upregulated production of these proteins, so that the overall glomerular expression did not decline despite reduced podocyte number.

Podocytes contain actin filaments in their foot processes. We addressed changes in podocyte/glomerular morphology by staining kidney sections of control and iPLA_2_γ KO mice for F-actin with fluorescein-conjugated (FITC)-phalloidin^28^. We did not detect any significant differences in phalloidin staining between groups (Supplementary Fig. 2). We did not examine podocyte foot processes or mitochondrial morphology by electron microscopy, since our previous experience suggested that this technique would not be sufficiently sensitive to demonstrate differences between untreated or adriamycin-treated control and KO mice in this age group^4^. In view of reduced podocyte numbers in adriamycin-treated KO mice, we also examined for activation of the mechanistic target of rapamycin (mTOR) pathway, which could potentially mediate hypertrophy of remaining podocytes. mTOR activity was monitored by phosphorylation of the mTOR substrate, S6 kinase, at Thr389^29,30^. There are typically two isoforms of S6 kinase (70 and 85 kDa). The 85 kDa isoform was expressed in glomeruli of adriamycin-treated mice, and showed greater phosphorylation in control mice, compared with iPLA_2_γ KO (Supplementary Fig. 3). We could not confidently identify the 70 kDa isoform in glomeruli. This result does not support mTOR activation as a pathway mediating hypertrophy in KO mice.

### Deletion of iPLA_2_γ enhances autophagy in adriamycin nephrosis

**Figure 3:**
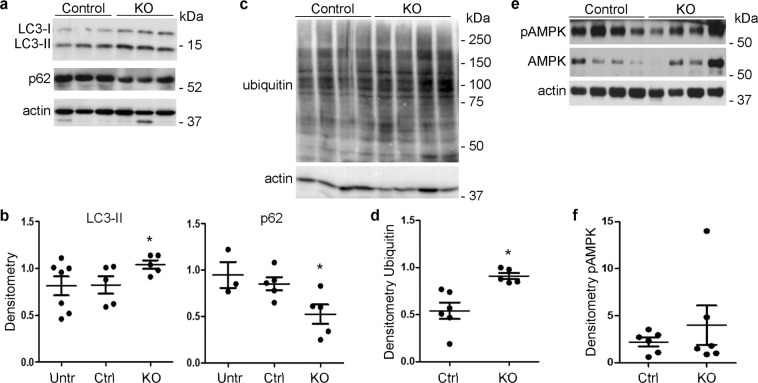
Deletion of iPLA_2_γ enhances autophagy and polyubiquitination in adriamycin nephrosis. Glomeruli were isolated from control (Ctrl) and iPLA_2_γ KO mice 4 weeks after adriamycin administration. (**a**,**c**,**e**) Representative immunoblots. Uncropped immunoblots are presented in Supplementary Fig. 5. (**b**,**d**,**f**) Densitometric quantification. (**a**,**b**) Glomerular lysates were immunoblotted with antibodies to LC3 and p62. LC3-II/actin was increased and p62/actin was decreased in iPLA_2_γ KO mice. *P < 0.035 KO vs control (adriamycin); 5 mice per group. In panel b, glomerular LC3-II levels in 6 untreated (Untr) mice (2 control and 4 KO) and p62 levels in 3 untreated mice (1 control and 2 KO) are shown for comparison. (**c**,**d**) Glomerular lysates were immunoblotted with anti-ubiquitin antibody. *P = 0.005 KO vs control, 6 control mice and 5 KO mice. (**e**,**f**) Lysates were immunoblotted with antibodies to pAMPK and AMPK. Levels of AMPK were highly variable among mice, and while there was an upward trend, there was not a significant difference in pAMPK/AMPK between control and KO mice (6 mice per group).

To address changes in metabolic pathways in adriamycin-induced podocyte injury and the role of iPLA_2_γ, isolated glomeruli were immunoblotted with antibodies to LC3 and p62^31^. The extent of lipidation of LC3 (i.e. conversion of LC3-I to LC3-II) or degradation of the autophagy-selective substrate, p62, can be used to monitor autophagy. Glomerular LC3-II was increased and p62 decreased in adriamycin-treated iPLA_2_γ KO mice, compared with treated control (Fig. 3a,b). Given that loss of iPLA_2_γ is associated with mitochondrial injury, these results are compatible with enhanced autophagy or mitophagy in the KO mice, presumably as a mechanism activated to clear damaged proteins or organelles. LC3-II and p62 levels in adriamycin-treated control mice were comparable to untreated WT and KO mice. It should be noted that in contrast to old mice^4^, at a young age (i.e. 3.5–4.5 months), untreated KO mice did not show significant differences in LC3-II or p62, compared with WT; therefore, these results have been presented together (Fig. 3a,b).

Misfolded of damaged proteins may be cleared not only by autophagy, but also via the ubiquitin-proteasome system^18^. Glomerular lysates of adriamycin-treated KO mice showed increased polyubiquitination of proteins, compared with treated control, implying enhanced ubiquitination and degradation of damaged proteins by the proteasome (Fig. 3c,d). To examine for evidence of mitochondrial dysfunction, we monitored phosphorylation (p) of AMP-activated protein kinase (AMPK) at Thr172^4^. Mitochondrial damage results in a decrease in ATP production, and reduced cellular levels of ATP stimulate phosphorylation of AMPK. Earlier, we showed that deletion of iPLA_2_γ increased pAMPK in aging mice, in keeping with mitochondrial dysfunction^4^. In the present study, glomerular AMPK expression was highly variable among control and KO mice treated with adriamycin, and while the mean pAMPK/AMPK level tended to be greater in KO mice, the increase above control was not statistically significant (Fig. 3e,f). The reason for the AMPK variability is unclear, but it appears expression of AMPK protein was affected by adriamycin to a variable extent among mice.

### Role of iPLA_2_γ in basal and ER stress-induced autophagy

**Figure 4:**
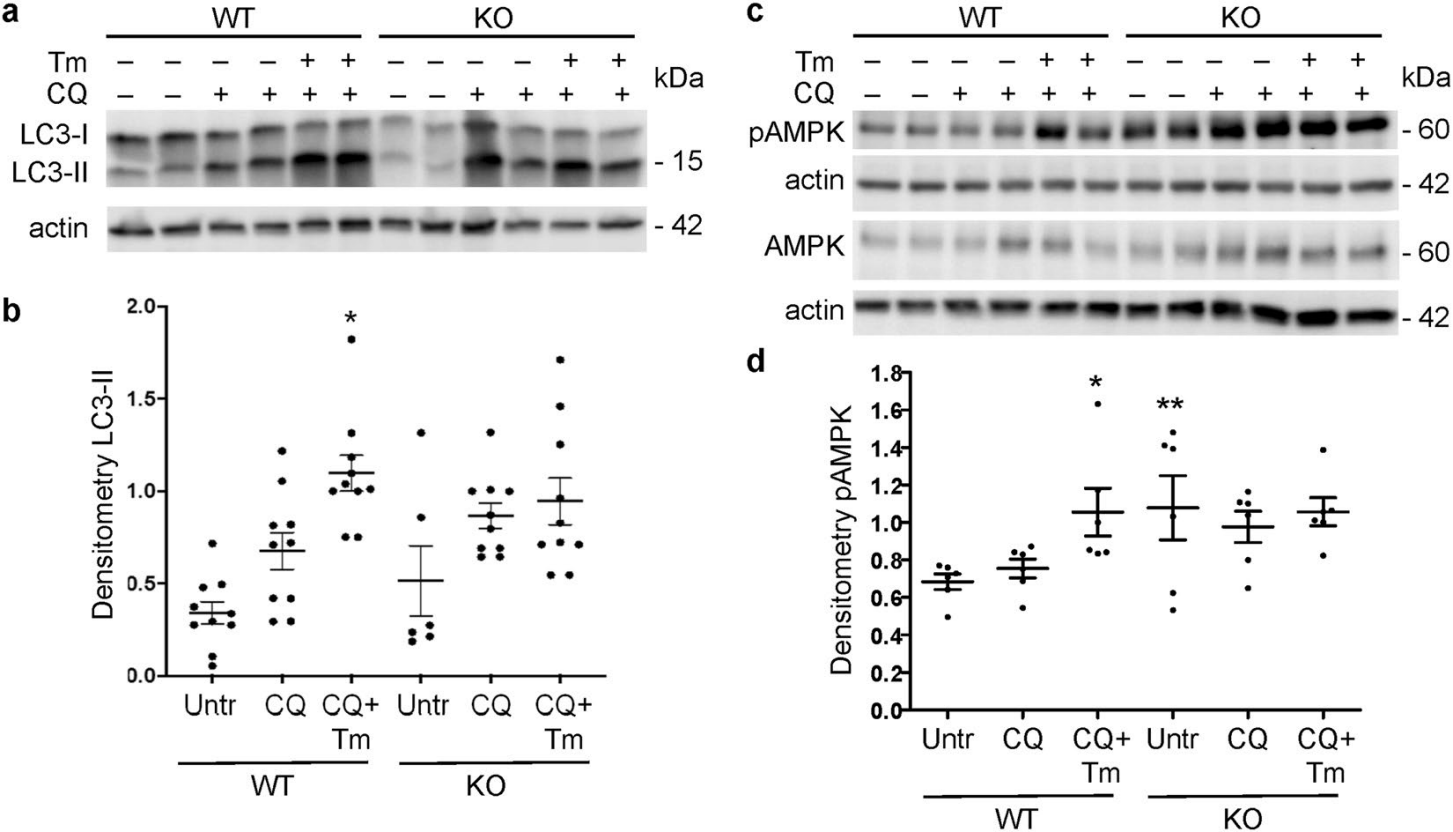
Role of iPLA_2_γ in basal and ER stress-induced changes in LC3 and pAMPK. WT and iPLA_2_γ KO GECs were untreated (Untr), or incubated with or without chloroquine (CQ, 25 µM) and tunicamycin (Tm, 5 µg/ml) for 18 h. Lysates were immunoblotted with antibodies to LC3 (**a**) or AMPK and pAMPK (**b**). (**a**,**c**) Representative immunoblots. Uncropped immunoblots are presented in Supplementary Fig. 5. (**b**,**d**) Densitometric quantification. (**b**) LC3-II/actin *P < 0.0001 KO vs WT (CQ + Tm), P = 0.08 KO vs WT (CQ). LC3-II/actin in KO/CQ was 146 ± 16% of WT/CQ (P < 0.01). 5 experiments performed in duplicate. (**d**) pAMPK/AMPK *P < 0.05 CQ + Tm vs Untr (WT), **P < 0.05 KO vs WT (Untr). 3 experiments performed in duplicate.

To further characterize the mechanistic role of iPLA_2_γ in podocyte injury, we turned to cultured GECs produced from WT and iPLA_2_γ KO mice. We examined the role of iPLA_2_γ in autophagy under basal and stressed conditions. WT and iPLA_2_γ KO GECs were treated with or without chloroquine. Chloroquine blocks the fusion of autophagosomes with lysosomes, which forms autolysosomes, and therefore the drug prevents autolysosomal protein degradation, allowing comparison of the rate of autophagosome formation. In the first set of experiments, GEC lysates were immunoblotted with anti-LC3 antibody. GECs were also treated with or without tunicamycin, which induces ER stress by causing accumulation of misfolded proteins in the ER and activates the unfolded protein response in these cells^8^. Under basal conditions (in the presence of chloroquine), LC3-II was greater in iPLA_2_γ KO cells, compared with WT (Fig. 4a,b), implying increased basal autophagy in KO cells, and in keeping with results of our earlier study where autophagy was increased significantly in KO cells^4^. Tunicamycin significantly enhanced LC3-II in WT GECs, and tended to increase LC3-II in iPLA_2_γ KO GECs, implying that ER stress-induced autophagy is facilitated by iPLA_2_γ (Fig. 4a,b).

**Figure 5:**
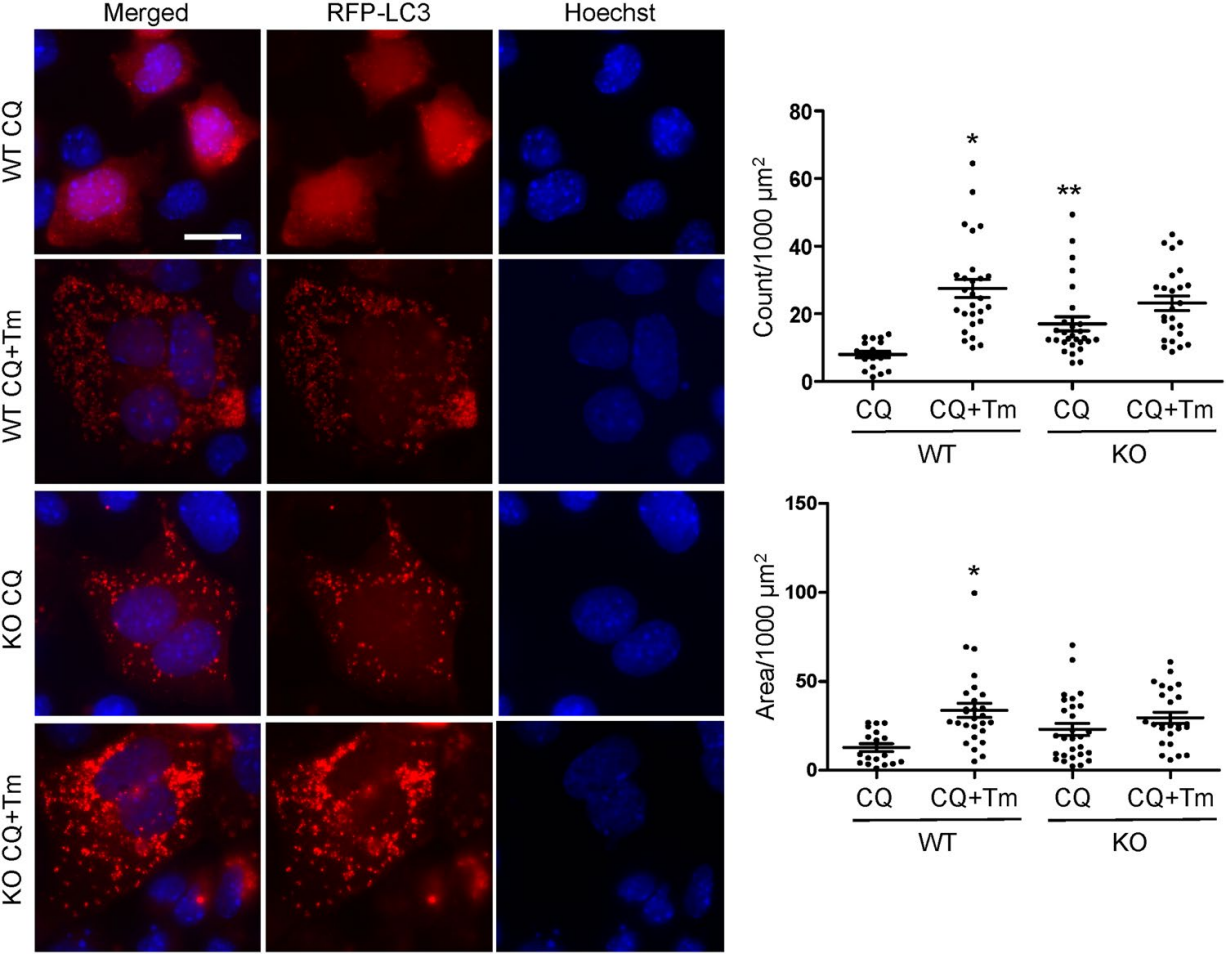
Role of iPLA_2_γ in basal and ER stress-induced changes in LC3-II puncta. WT and iPLA_2_γ KO GECs were transfected with RFP-LC3, and were incubated with or without chloroquine (CQ, 25 µM) and tunicamycin (Tm, 5 µg/ml) for 18 h. Representative photomicrographs and quantification of LC3-II puncta are presented. Bar = 25 µm. Puncta count: *P < 0.001 CQ + Tm vs CQ (WT), **P < 0.05 KO vs WT (CQ). Puncta area: *P < 0.001 CQ + Tm vs CQ (WT). 18–28 cells per group in 2 experiments.

Conversion of LC3-I to LC3-II in cultured GECs was also addressed by monitoring the formation of LC3-II puncta by fluorescence microscopy. In this assay, a low level of autophagy is reflected by red fluorescent protein (RFP)-LC3 mainly in the cytosol in the form of LC3-I. An increase in autophagy is revealed by formation of puncta, reflecting conversion of LC3-I to LC3-II and accumulation of LC3-II in autophagosomes^4^. GECs, transfected with RFP-LC3, were treated with chloroquine and/or tunicamycin. Quantification of the puncta demonstrated that resting WT cells treated with chloroquine contained relatively few puncta and that the puncta constituted only a minimal proportion of cell area (Fig. 5). RFP-LC3-II puncta and puncta area were markedly enhanced in KO cells (in the presence of chloroquine), consistent with the formation of autophagosomes (Fig. 5). Tunicamycin treatment (in the presence of chloroquine) significantly increased the number of puncta in WT cells, thus confirming enhanced autophagy. The number of RFP-LC3-II puncta in iPLA_2_γ KO cells after treatment with tunicamycin tended to increase, compared with KO cells, but the change was not significant. Thus, the assay confirmed that there is increased basal autophagy in iPLA_2_γ KO cells and that ER stress-induced autophagy is facilitated by iPLA_2_γ.

### Deletion of iPLA_2_γ results in mitochondrial dysfunction

To address the effect of iPLA_2_γ on mitochondrial function, we monitored phosphorylation of AMPK, a sensor of cellular ATP levels. AMPK phosphorylation in GECs was investigated under basal conditions and during ER stress. Basal phosphorylation of AMPK in lysates of iPLA_2_γ KO cells was significantly greater than in WT cells (Fig. 4c,d). Furthermore, compared with WT cells, iPLA_2_γ KO GECs showed reduced basal and maximal oxygen consumption rates (OCR) (Supplementary Fig. 4). The decrease in the maximal respiration in KO GECs is in support of mitochondrial dysfunction and reduced capacity for ATP production. These results are in keeping with our previous observation that in iPLA_2_γ KO GECs, there was significantly decreased MitoTracker Red CMXRos staining (which reflects reduced mitochondrial membrane potential) compared with WT cells^4^. Together, the results indicate that deletion of iPLA_2_γ induces mitochondrial dysfunction.

Tunicamycin increased phosphorylation of AMPK in WT cells significantly, compared with untreated cells (Fig. 4c,d). In contrast, tunicamycin (at the same dose) did not enhance pAMPK further in iPLA_2_γ KO cells. Interestingly, tunicamycin, besides inducing ER stress, appears to induce mitochondrial dysfunction in WT GECs, although no additional exacerbation of mitochondrial dysfunction was evident in KO cells.

### Effect of iPLA_2_γ, adriamycin and carbonyl cyanide m-chlorophenylhydrazone (CCCP) on pAMPK and LC3-II

**Figure 6:**
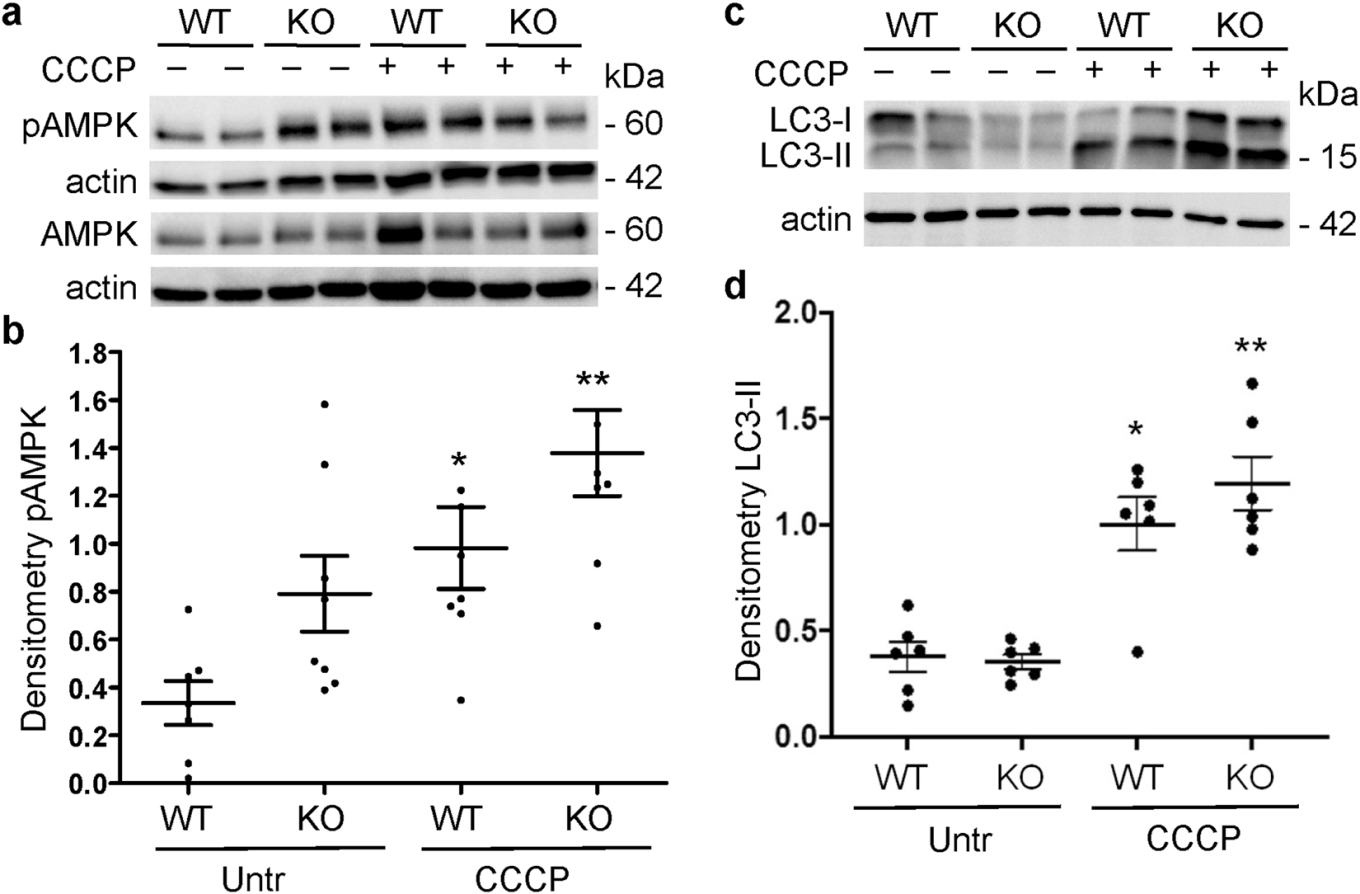
Effect of CCCP on pAMPK and LC3. WT and iPLA_2_γ KO GECs were untreated (Untr), or incubated with CCCP (10 µM) for 18 h. Lysates were immunoblotted with antibodies to AMPK and pAMPK (**a**) or LC3 (**b**). (**a**,**c**) Representative immunoblots. Uncropped immunoblots are presented in Supplementary Fig. 5. (**b**,**d**) Densitometric quantification. (**b**) pAMPK/AMPK *P < 0.05 CCCP vs Untr (WT), **P < 0.05 CCCP vs Untr (KO). 3 experiments performed in duplicate. (**d**) LC3-II/actin *P = 0.001 CCCP vs Untr (WT), **P < 0.0001 CCCP vs Untr (KO). 3 experiments performed in duplicate.

In these experiments we addressed the effects of iPLA_2_γ on mitochondrial function and autophagy in the presence or absence of adriamycin. For comparison, we also studied the effect of another exogenous stimulus, CCCP, which causes an uncoupling of the proton gradient in the mitochondrial electron transport chain and inhibits oxidative phosphorylation. In keeping with the results presented above (Fig. 4), phosphorylation of AMPK was increased in iPLA_2_γ KO cells, compared with WT, consistent with reduced ATP generation by mitochondria (Fig. 6a,b). In addition, disruption of mitochondrial function in WT and iPLA_2_γ KO GECs with CCCP significantly increased pAMPK, compared with the corresponding untreated cells (Fig. 6a,b). Adriamycin increased pAMPK in KO GECs (215 ± 56% of control, P = 0.015), but there was no significant change in WT cells (80 ± 10% of control, 4 experiments performed in duplicate or triplicate).

We also examined the effects of CCCP and adriamycin on LC3-II, using immunoblotting. Treatment of WT and KO GECs with CCCP resulted in a significantly greater increase in LC3-II, compared with untreated cells (Fig. 6c,d). By analogy, adriamycin (18 h incubation) increased LC3-II in WT GECs (348 ± 61% of control, P < 0.005), as well as in KO cells (325 ± 104% of control, P < 0.04; 4 experiments performed in duplicate or triplicate). In addition, adriamycin reduced the basal and maximal OCR in WT GECs (Supplementary Fig. 4). There was no further reduction in the OCR in adriamycin-treated KO cells, compared with untreated KO cells, where the OCR was already markedly reduced (expressed per 1,000 cells); however, when analyzed as percent of basal OCR, then the maximal OCR was also further reduced in adriamycin-treated KO cells, consistent with mitochondrial dysfunction (Supplementary Fig. 4).

### Deletion of iPLA_2_γ induces mitophagy

**Figure 7:**
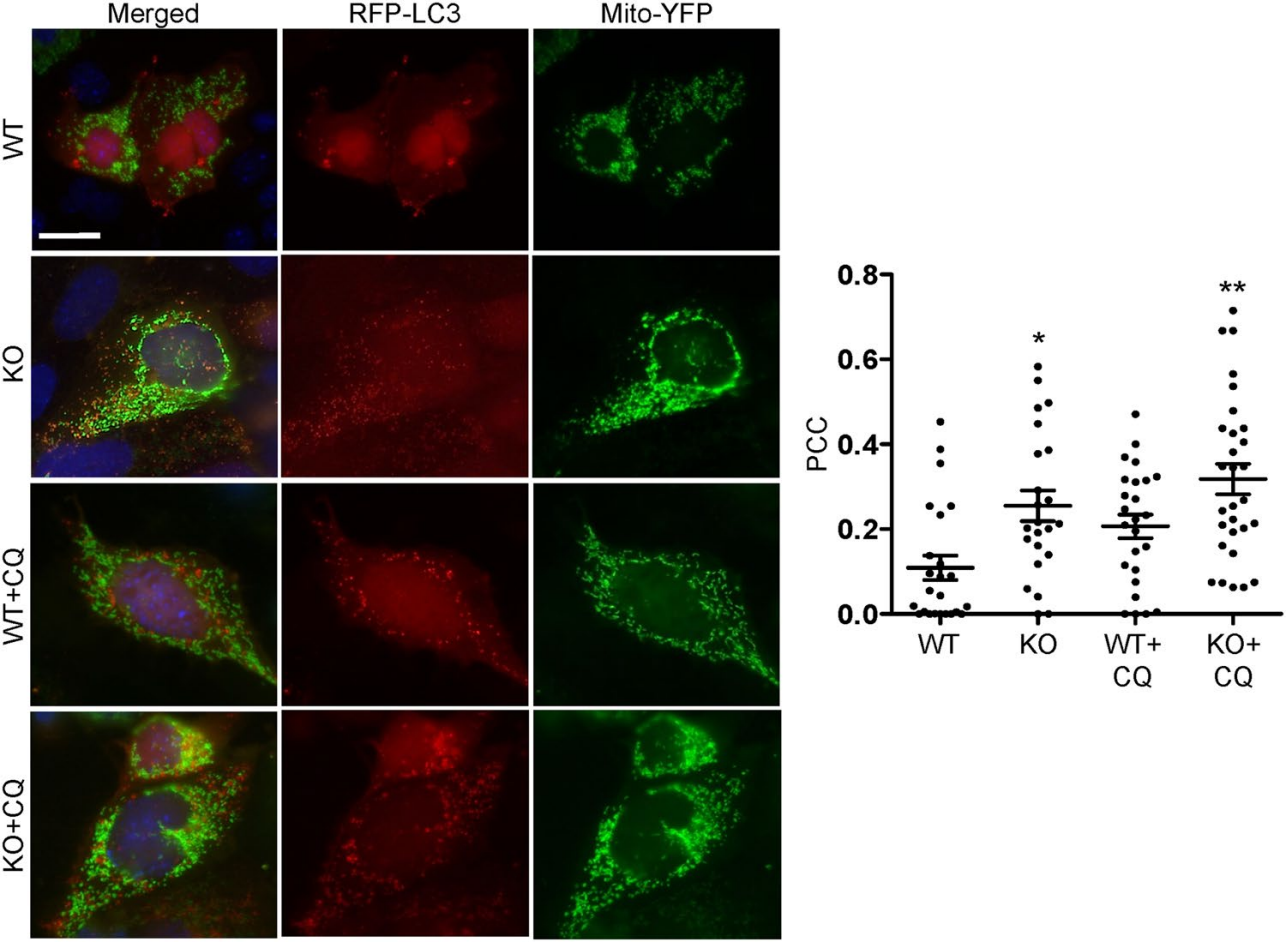
Effect of iPLA_2_γ on delivery of mitochondria to autophagosomes. WT and iPLA_2_γ KO GECs were co-transfected with RFP-LC3 and mito-YFP cDNAs. Cells were then incubated with or without chloroquine (CQ) for 6 h. Representative photomicrographs and the Pearson correlation coefficient (PCC) for the colocalization of RFP-LC3 and mito-YFP are presented. Bar = 25 µm. *P < 0.01 KO vs WT, **P < 0.05 KO + CQ vs WT + CQ. 23–29 cells per group in 4 experiments.

The above experiments prompted us to examine if the enhanced autophagy in iPLA_2_γ KO GECs consisted at least in part of mitophagy. In the first set of experiments, we monitored mitophagy by examining colocalization of RFP-LC3-II, an autophagy marker, with mito-yellow fluorescent protein (YFP), a mitochondrial marker, using fluorescence microscopy. Colocalization of RFP-LC3-II and mito-YFP would denote mitochondria within autophagosomes^19,20^. WT and iPLA_2_γ KO GECs were co-transfected with RFP-LC3 and mito-YFP cDNAs. Cells were then incubated with or without chloroquine. In absence of chloroquine, RFP-LC3 was found in the cytosol of WT GECs in the form of LC3-I, while in iPLA_2_γ KO GECs, RFP-LC3 was punctate, reflecting conversion of LC3-I to LC3-II and accumulation of LC3-II in autophagosomes (Fig. 7). Colocalization of LC3-II and mito-YFP was observed as yellow-orange puncta in iPLA_2_γ KO GECs, and was quantified by the Pearson correlation coefficient. In the absence of chloroquine, there was minimal colocalization of LC3-II and mito-YFP in WT cells, but colocalization of LC3-II and mito-YFP was significantly greater in KO cells (Fig. 7). In the presence of chloroquine, colocalization of LC3-II and mito-YFP increased in WT cells, compared with untreated, and there was a further increase in KO cells. The greater colocalization of RFP-LC3-II with mito-YFP in puncta in the KO cells is in keeping with enhanced mitophagy. However, it should be noted that the greatest Pearson correlation coefficient was ~0.3, indicating that the majority of RFP-LC3 and mito-YFP fluorescence was not colocalized, i.e. only a minority of mitochondria in the cells were undergoing mitophagy.

**Figure 8:**
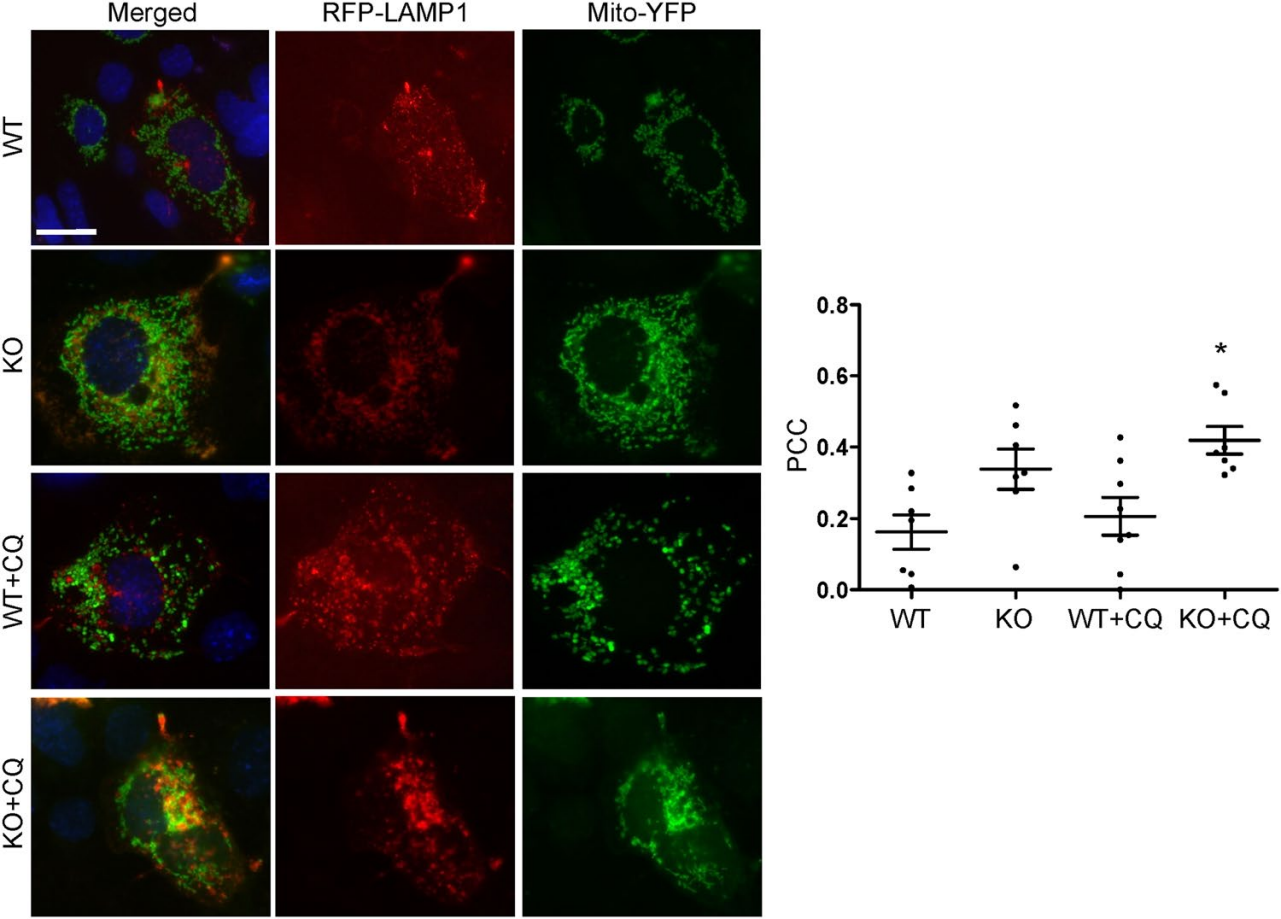
Effect of iPLA_2_γ on delivery of mitochondria to lysosomes. WT and iPLA_2_γ KO GECs were co-transfected with RFP-LAMP1 and mito-YFP cDNAs. Cells were then incubated with or without chloroquine (CQ) for 6 h. Representative photomicrographs and the Pearson correlation coefficient (PCC) for the colocalization of RFP-LAMP1 and mito-YFP are presented. Bar = 25 µm. *P < 0.05 KO + CQ vs WT + CQ. 7–8 cells per group in 2 experiments.

Second, we used a complementary fluorescence technique to monitor the delivery of the mitochondria to the lysosomes^19,20^. WT and iPLA_2_γ KO GECs were co-transfected with cDNAs encoding RFP-lysosomal-associated membrane protein-1 (LAMP1) and mito-YFP. Similar to the above results, colocalization of RFP-LAMP-1 and mito-YFP was observed in iPLA_2_γ KO cells, and was significantly increased in presence of chloroquine, compared with WT cells in presence of chloroquine (Fig. 8). Colocalization of RFP-LAMP-1 and mito-YFP tended to be greater in KO cells, compared with WT in the absence of chloroquine. These results imply that deletion of iPLA_2_γ induces mitophagy and the autophagosomes containing the mitochondria are delivered to lysosomes by forming autolysosomes. There was a slight increase in RFP-LAMP-1 and mito-YFP colocalization in chloroquine-treated KO cells, compared with untreated KO cells (Fig. 8). This may reflect inhibition of lysosomal degradation, although the effect might be offset by blockade of autophagosomal fusion.

### Induction of mitophagy by adriamycin and CCCP

**Figure 9:**
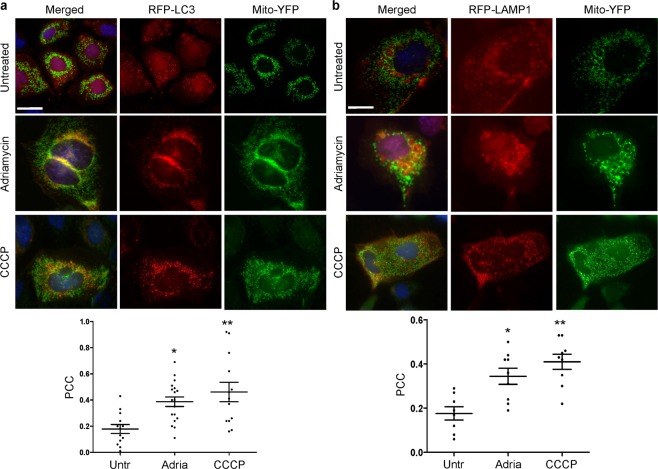
Induction of mitophagy by adriamycin and CCCP. WT GECs were co-transfected with RFP-LC3 and mito-YFP (**a**) or RFP-LAMP1 and mito-YFP cDNAs. (**b**) Cells were then untreated (Untr), or incubated with CCCP (10 µM) or adriamycin (Adria; 1 µM) in the presence of chloroquine (CQ) for 24 h. Representative photomicrographs and the Pearson correlation coefficient (PCC) for colocalization are presented. Bars = 25 µm. (**a**) *P < 0.05 Adriamycin vs Untreated, **P < 0.01 CCCP vs Untreated. 13–18 cells per group in 3 experiments. (**b**) *P < 0.01 Adriamycin vs Untreated, **P < 0.001 CCCP vs Untreated. 7–8 cells per group in 2 experiments.

Since adriamycin enhanced podocyte injury in iPLA_2_γ KO mice, we examined the effect of adriamycin on mitophagy in cultured GECs. For comparison, we also studied the effect of CCCP, which targets mitochondria directly. CCCP or adriamycin treatment for 24 h induced mitophagy, which was shown by ~2–3-fold increase in colocalization of LC3-II and mito-YFP in WT GECs, in presence of chloroquine (Fig. 9a). CCCP tended to be more robust in inducing mitophagy than adriamycin, but the difference was not statistically significant. Similarly, both CCCP and adriamycin induced colocalization of RFP-LAMP-1 and mito-YFP in chloroquine-treated WT GECs (Fig. 9b). We also examined the effect of CCCP on mitophagy in iPLA_2_γ KO cells (protocol as in Fig. 9a in WT cells). In these experiments, the Pearson correlation coefficient for colocalization of LC3-II and mito-YFP was 0.41 ± 0.04 in untreated KO cells and 0.42 ± 0.03 after CCCP treatment (16–38 cells per group in 2 experiments). For comparison, in WT cells that were treated with CCCP in parallel, the Pearson correlation coefficient was 0.52 ± 0.08. Thus, the basal Pearson correlation coefficient in iPLA_2_γ KO cells (basal mitophagy) was substantially greater than the basal value in WT cells shown in Fig. 9a (0.18 ± 0.03), and CCCP did not induce a further increase in mitophagy in the KO cells. Taken together, the above results suggest that when compared with WT GECs, deletion of iPLA_2_γ in GECs induces mitophagy (Figs. 7 and 8) to a comparable magnitude as treating WT GECs with CCCP or adriamycin (Fig. 9).

## Discussion

Aging iPLA_2_γ KO mice show ultrastructural injury to podocyte plasma membranes and mitochondria, as well as an increased number of autophagosomes in podocytes, although these mice do not develop albuminuria^4^. Interestingly, although deletion of iPLA_2_γ in this earlier study was global, there were no apparent changes in mitochondrial ultrastructure nor in the number of autophagosomes in glomerular mesangial and endothelial cells. In the present study, we show that deletion of iPLA_2_γ results in albuminuria and podocyte depletion in young mice with chronic adriamycin nephrosis, an experimental model of FSGS, whereas mice that express iPLA_2_γ were resistant to adriamycin-induced injury (Figs 1 and 2). Thus, in the absence of iPLA_2_γ, podocyte injury was unmasked when these cells were stressed. Furthermore, compared with adriamycin-treated control mice, glomerular LC3-II was increased and p62 decreased in treated iPLA_2_γ KO mice, in keeping with increased autophagy (Fig. 3). Deletion of iPLA_2_γ also increased glomerular polyubiquitination of proteins (Fig. 3). Together, the enhanced autophagy and ubiquitination support the view that these pathways are activated to clear damaged or misfolded proteins that accumulate in podocytes in adriamycin nephrosis. Indeed, in Atg5 KO mice, autophagy was shown to be a homeostatic mechanism that maintains podocyte integrity, and may protect against aging and glomerular injury^16^. Autophagy may facilitate cell survival by improving energy levels, and clearing damaged mitochondria from the cytoplasm (see below)^16–18,21^. Whether the proteasome or crosstalk between autophagy and the ubiquitin-proteasome is an important mechanism in adriamycin-induced injury requires further study. However, in contrast to the situation in adriamycin nephrosis, we previously showed no differences in polyubiquitination of glomerular proteins between aging WT and iPLA_2_γ KO mice, despite enhanced autophagy^4^.

Based on the observations that autophagy is upregulated in adriamycin nephrosis (Fig. 3) and in aging iPLA_2_γ KO mice^4^, we turned to cultured GECs to further address mechanisms. Previously, we demonstrated that in WT GECs, GFP-tagged iPLA_2_γ is localized at the ER and mitochondria, and localization was dependent on the N-terminal domain of iPLA_2_γ^6^. In the present study, we confirmed that basal autophagy is enhanced in GECs derived from iPLA_2_γ KO mice, compared with WT cells (Figs 4 and 5). However, ER stress-induced autophagy was more pronounced in the WT cells. iPLA_2_γ KO cells showed decreased maximal OCR (Supplementary Fig. 4) and increased phosphorylation of AMPK (Fig. 4). This result is consistent with our previous study, in which we showed that phosphorylation of AMPK was increased in glomeruli isolated from aging iPLA_2_γ KO mice, and with results in cultured GECs showing mitochondrial dysfunction in iPLA_2_γ KO cells^4^. Treatment of WT and iPLA_2_γ KO GECs with adriamycin increased both LC3-II and AMPK phosphorylation, mainly in KO GECs. Similarly, an upward trend in pAMPK was evident in glomeruli of iPLA_2_γ KO mice; however, levels of AMPK protein were highly variable among mice treated with adriamycin, precluding precise comparisons of pAMPK/AMPK ratios among these mice (Fig. 3). As expected, CCCP, which inhibits oxidative phosphorylation in mitochondria and thereby lowers ATP levels, stimulated AMPK phosphorylation in WT and iPLA_2_γ KO GECs (Fig. 6). In addition, CCCP enhanced LC3-II in GECs (Fig. 6). Together, these experiments support the view that deletion of iPLA_2_γ results in mitochondrial dysfunction and reduces cellular ATP, stimulates AMPK, and enhances autophagy. The latter may, at least in part, compensate for impaired energy homeostasis. Furthermore, although adriamycin can potentially activate several cytotoxic pathways in cells, this drug can exacerbate ATP depletion, AMPK activation, and autophagy in the context of iPLA_2_γ deletion. Indeed, AMPK was shown to be an important positive regulator of autophagy^16,17^.

The presence of mitochondrial damage and increased LC3-II levels in iPLA_2_γ KO GECs and glomeruli, as well as increases in LC3-II induced by adriamycin, suggested that deletion of iPLA_2_γ was inducing not only autophagy, but also mitophagy. A viable pool of mitochondria in cells is maintained by biogenesis and degradation. The latter removes defective mitochondria and occurs principally through mitophagy^19,21^. There was increased colocalization of RFP-LC3-II and mito-YFP in iPLA_2_γ KO GECs (denoting mitochondria within autophagosomes), compared with WT (Fig. 7). Similarly, colocalization of RFP-LAMP-1 and mito-YFP (reflecting delivery of mitochondria to lysosomes) was enhanced in iPLA_2_γ KO cells (Fig. 8). Treatment of GECs with adriamycin or CCCP resulted in increased colocalization of RFP-LC3-II and mito-YFP, as well as RFP-LAMP-1 and mito-YFP (Fig. 9). Therefore, mitophagy was increased in iPLA_2_γ KO GECs, as well as in WT GECs treated with adriamycin and CCCP, the latter providing verification linking mitochondrial dysfunction with mitophagy in these cells. It should, however, be noted that the Pearson correlation coefficient in KO cells, and in cells treated with adriamycin and CCCP was in the range of 0.3–0.4, indicating only moderate colocalization, which implies that in these cells, the majority of mitochondria were not within autophagosomes or lysosomes, and were probably functional.

Our results in podocytes and adriamycin nephrosis are consistent with other studies, showing that deletion of iPLA_2_γ in mice caused mitochondrial disruption. KO of iPLA_2_γ altered mitochondrial phospholipid homeostasis, specifically, decreased cardiolipin content, and changed the distribution of cardiolipin molecular species in mitochondria of the heart and skeletal muscle^10,13,32^. iPLA_2_γ KO mice showed an increase in various cardiolipin molecular species in the brain, as well as changes in levels of other lipids^11^. Proper cardiolipin content and its remodeling is required for efficient function of the mitochondrial electron transport chain, and abnormal remodeling in iPLA_2_γ KO tissues may result in bioenergetic inefficiency^11^. Defective iPLA_2_γ-mediated release of fatty acids may also impair signaling that is required for mitochondrial function^32^. iPLA_2_γ KO mice demonstrate mitochondrial dysfunction in various organs, such as the heart, skeletal muscle, liver and brain, particularly in aging mice^10,11,13,32^. These mice show growth retardation, cold intolerance, bioenergetic dysfunctional phenotypes, oxidative stress, and lipid peroxidation^10,13^. Together, the various studies demonstrate an important role for iPLA_2_γ in mitochondrial structure and function.

By analogy to our results in adriamycin nephrosis (a chronic proteinuric glomerulopathy), the induction of podocyte injury in acute complement-mediated heterologous anti-GBM nephritis in young mice also showed a protective role for iPLA_2_γ^4^. iPLA_2_γ KO mice with anti-GBM nephritis showed enhanced albuminuria, depletion of podocytes, and podocyte injury, compared with controls. Deletion of mitochondrial genes, e.g. Mpv17, leads to glomerular abnormalities, and worsens injury in anti-GBM nephritis^22,33^. In humans, mutations in mitochondrial genes can result in hereditary FSGS^34,35^. Mitochondrial dysfunction is becoming increasingly recognized as contributing to experimental and human glomerular diseases, including congenital nephrotic syndrome, acquired FSGS and diabetic nephropathy^36–39^. Thus, intact mitochondrial function is essential to podocyte homeostasis, and iPLA_2_γ plays an important role in the maintenance of podocyte integrity both in health and disease. Understanding the mechanisms by which iPLA_2_γ maintains mitochondrial structure and function, and how damaged mitochondria may be repaired in the glomerulus is essential for development of novel therapies for glomerular disease and proteinuria. In the future, targeting the enzymatic activity of iPLA_2_γ may be a new approach to limit podocyte injury and proteinuria.

## Materials and Methods

### Materials

Tissue culture media and Lipofectamine 2000 were purchased from Wisent (Saint-Jean-Baptiste, QC) and Invitrogen-Life Technologies (Burlington, ON). Electrophoresis reagents were purchased from Bio-Rad Laboratories (Mississauga, ON), and GE Healthcare (Baie d’Urfé, QC). Adriamycin, tunicamycin, chloroquine, CCCP and FITC-phalloidin were purchased from Sigma-Aldrich (St. Louis, MO). Rabbit anti-WT1 (192), goat anti-synaptopodin (21537) and rabbit anti-S6 kinase (C-18, sc-230) antibodies were purchased from Santa Cruz Biotechnology (Santa Cruz, CA). Goat anti-podocalyxin antibody (AF1556) was from R & D Systems (Minneapolis, MN). Rabbit antibodies to LC3B (2775), phospho-AMPK-α (Thr172; 2531), AMPKα (2532), p62 (SQSTM1; 5114), and phospho-S6 kinase (Thr389; 9205) were from Cell Signaling Technology (Danvers, MA). Rabbit anti-ubiquitin antibody (U5379) was from Sigma. Rabbit anti-nephrin antiserum was kindly provided by Dr. Tomoko Takano (McGill University), and was characterized previously^4,40^. Plasmid mito-YFP (cytochrome c oxidase-subunit IV fused with YFP; 10089272) was from American Type Culture Collection (Manassas, VA). Plasmid RFP-LAMP1 (1817) and pmRFP-LC3 (21075) were from Addgene^41,42^.

### Mice

iPLA_2_γ KO mice in a C57BL/6 background were kindly provided by Dr. Richard Gross (Washington University, St. Louis, MO, USA). Mice were produced, bred and genotyped, as described previously^4,10^. Mice were housed in a standard animal care facility with 12 h on-off light cycles, and were fed ad libitum. Adriamycin nephrosis was induced by a single tail-intravenous injection of adriamycin (12 mg/kg)^23^. Urine was collected at weekly intervals in the morning. After 4 weeks, kidneys were collected for IF microscopy, and glomeruli were isolated utilizing a differential sieving technique^4^. Metabolic parameters in iPLA_2_γ KO mice have been reported previously^43^. KO mice have lower weights compared with WT, with the differences becoming statistically significant at 4.5 months. The two groups of mice have similar fasting serum glucose and cholesterol levels, and similar food consumption, dietary fat digestion and absorption^43^. Animal protocols were reviewed and approved by the McGill University Animal Care Committee. All methods were performed in accordance with the relevant guidelines and regulations. Mouse urine albumin concentration was quantified using an enzyme-linked immunosorbent assay kit (Bethyl Laboratories. Montgomery, TX), as described previously^4^. Urine creatinine concentration was measured using a colorimetric assay kit (Cayman Chemical Company, Ann Arbor, MI), as described previously^4^.

### Cell culture and transfection

GECs were derived from iPLA_2_γ KO mice and wild type (WT) control mice. The detailed method and characterization of the cells was published previously^8^. WT and iPLA_2_γ KO cell lines were cultured on plastic substratum in K1 medium (DMEM, Ham F-12, with 5% NuSerum and hormone mixture) and were co-transfected with plasmids encoding RFP-LC3 and mito-YFP, or plasmids RFP-LAMP1 and mito-YFP, using Lipofectamine 2000, according to the manufacturer’s instructions^4,8^.

### Immunofluorescence microscopy

IF microscopy using frozen kidney sections was described previously^4^. WT and iPLA_2_γ KO cells were seeded on coverslips and were transiently co-transfected with plasmids encoding cDNAs fused with fluorescent reporters^4^. At 24 h after transfection, cells were either untreated or treated with stimulus, fixed with paraformaldehyde (4% in PBS), and then stained with Hoechst H33342 and rinsed with ice cold PBS before being mounted on glass slides. Stained kidney sections and cells were examined with a Zeiss Axio Observer fluorescence microscope with visual output connected to an AxioCam MRm monochrome camera. To allow comparisons of fluorescence intensity, all images were taken at the same exposure. Final images were collected from series of images derived from different focal planes (Z-stack) using AxioVision 4.8 software. WT1-positive cells were quantified by visual counting^4^. Fluorescence intensity of kidney sections and autophagic puncta (the number of puncta and total area of the puncta) was quantified using ImageJ software, as described previously^4^. Autophagic puncta were defined as structures having areas within the set range of 0.2–25 µm^2^. Colocalization of fluorescent signals was measured by the Pearson correlation coefficient using ImageJ and the Coloc module. The method used in this module is called the “thresholding method” and the thresholded Pearson’s correlation coefficient was calculated according to Adler and Parmyrd, using only intensity values over a determined threshold in both channels^44^.

### Immunoblotting

Isolated glomeruli or cells were lysed in ice-cold buffer containing 1% Triton X-100, 125 mM NaCl, 10 mM Tris, pH 7.4, 1 mM EGTA, 2 mM Na_3_VO_4_, 10 mM sodium pyrophosphate, 25 mM NaF, and protease inhibitor mixture. The lysates were then centrifuged at 13,000 g for 10 min. Lysate proteins were dissolved in Laemmli buffer and were subjected to SDS-PAGE under reducing conditions. Proteins were electrophoretically transferred to polyvinylidene difluoride membrane and blocked at room temperature for 1 h with 5% BSA and incubated with primary antibody followed by horseradish peroxidase-conjugated secondary antibody. Membranes were then developed with ECL, using several exposures to ensure that densities of signals were within a linear range, and to prevent saturation. Quantitative densitometry was performed using ImageJ^4,8^. Data were normalized within each experiment^45^, and results are presented in arbitrary units.

### Measurement of the oxygen consumption rate (OCR)

OCR measurements were performed on a Seahorse XFe96 extracellular flux analyzer using the Seahorse XF Cell Mito Stress Test kit (Agilent, Santa Clara, CA) according to the manufacturer’s instructions and standard protocol^46^. Briefly, GECs were seeded on XFe96 plates at 20,000, 50,000, or 100,000 cells per well, and incubated with or without adriamycin (1 µM) for 24 h. The OCR was measured under basal conditions and after exposure to mitochondrial modulators in the following order: oligomycin (1 µM, which inhibits ATP synthase and reduces OCR), carbonyl cyanide 4-trifluoromethoxyphenylhydrazone (FCCP, 1.5 µM, which uncouples oxygen consumption from ATP production and raises OCR to a maximal value), and a combination of rotenone and antimycin A (both at 0.5 µM, which reduce OCR to a minimal value), as described^46^. After the OCR readings were completed, cells were fixed in 4% paraformaldehyde and stained with a 0.05% crystal violet solution for 30 min. Next, GECs were washed and lysed in 1% SDS. Absorbance of each well (which reflects cell number) was read at 595 nm using an Infinite 200 Pro plate reader (Tecan Group Ltd, Männedorf, Switzerland). Since the purpose of this study was to address the effect of iPLA_2_γ on mitochondrial respiration, we calculated the basal OCR and maximal OCR (i.e. after uncoupling with FCCP)^46^. OCR values were normalized to cell number, and maximal OCR was also expressed as percent of respective basal OCR or percent of control (i.e. untreated WT cells).

### Statistics

Individual data points, as well as mean ± SE are presented. One- or two-way ANOVA was used to determine significant differences among groups. Where significant differences were found, individual comparisons were made between groups using the Student’s t-test and adjusting the critical value according to Bonferroni’s method (post hoc analysis). The Student’s t-test was used to determine significant differences between two groups.

## Supplementary information

Supplementary Information

## Data Availability

The data of the current study are available from the corresponding author on reasonable request.
