# Supplementary material for: Genetic Ablation of Calcium-independent Phospholipase A2γ Exacerbates Glomerular Injury in Adriamycin Nephrosis in Mice
Source: Sci Rep. 2019 Nov 7;9:16229. doi: 10.1038/s41598-019-52834-x (PMC6838178; doi:10.1038/s41598-019-52834-x)

Genetic Ablation of Calcium-independent Phospholipase $A_2\gamma$ Exacerbates Glomerular Injury in Adriamycin Nephrosis in Mice

Hanan Elimam, Joan Papillon, Julie Guillemette, José R. Navarro-Betancourt, and Andrey V. Cybulsky

Supplementary Information

Supplementary Figure 1. Deletion of iPLA$_2$γ exacerbates albuminuria in adriamycin nephrosis. Data in Figure 1 are presented in log-transformed format. *P<0.0001 KO vs control (Ctrl).

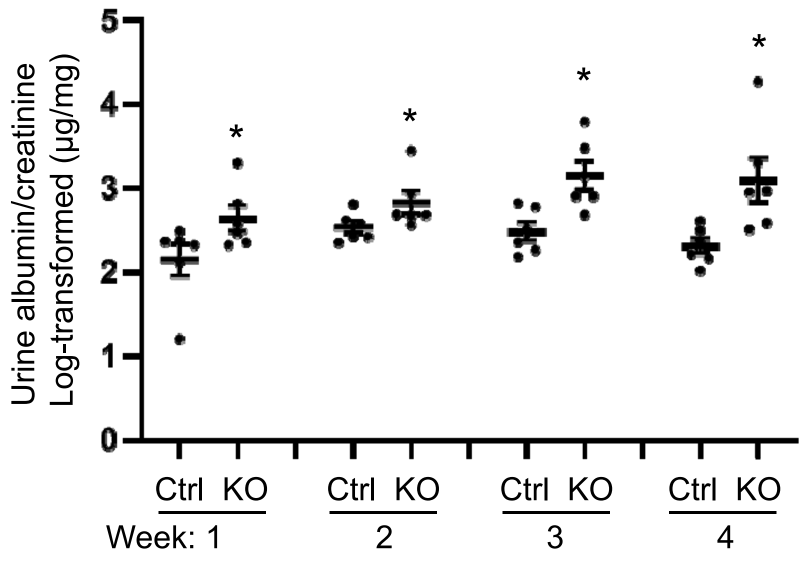

Supplementary Figure 2. F-actin in mouse glomeruli. Kidney sections were stained with FITC-phalloidin. There were no significant differences in phalloidin staining between groups. Bar = 25 µm.

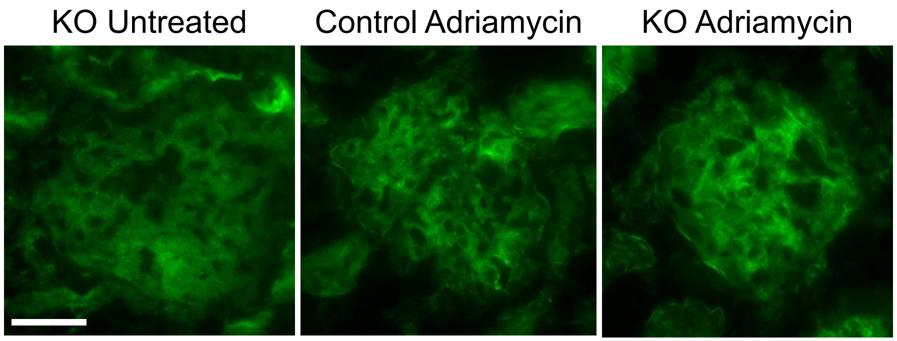

Supplementary Figure 3. Phosphorylated S6 kinase, 85 kDa isoform (pp85) is reduced in glomeruli of adriamycin-treated iPLA$_2\gamma$ KO mice, compared with control. Glomerular epithelial cells treated with or without the mTOR inhibitor, rapamycin (Rapa), are shown for comparison (4 lanes on the right). In the cells, lanes 3 and 4 show a lower exposure of lanes 1 and 2. S6 kinase phosphorylation is present basally, and as expected, is abolished after incubating cells with rapamycin. Densitometric quantification of pp85 is shown below the immunoblots. C, control/adriamycin; KO, knockout/adriamycin.

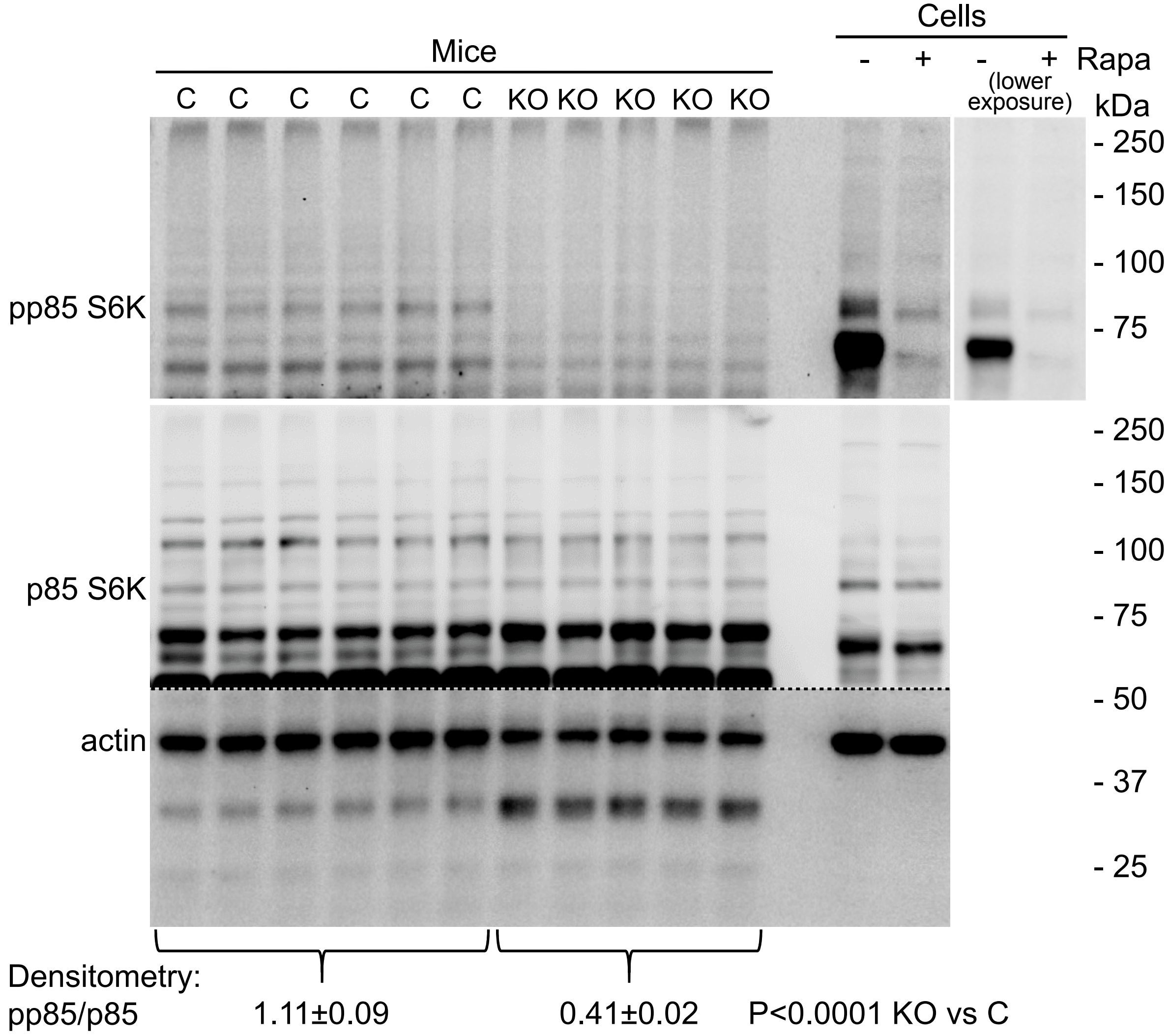

Supplementary Figure 4. Maximal oxygen consumption rate (Max OCR) is reduced in iPLA$_2\gamma$ KO glomerular epithelial cells (GECs). WT (control) and iPLA$_2\gamma$ KO GECs were plated at 100,000 cells per well. Then, cells were untreated or treated with adriamycin (1 μM) for 24 h. Subsequently, GECs underwent the Seahorse XF Cell Mito Stress Test on a Seahorse XFe96 extracellular flux analyzer and OCR readings were normalized to cell number.
Basal OCR was established in the absence of mitochondrial modulators (a). Max OCR was measured after uncoupling with FCCP (1.5 μM) (b). Max OCR values are also presented as a percentage of the respective basal OCR (c), and as a percentage of the maximal OCR in the control group (i.e. untreated WT GECs) (d). a) *$P<0.001$ KO vs WT (untreated). b) *$P<0.001$ KO vs WT (untreated), **$P<0.05$ KO vs WT (adriamycin). c) *$P<0.05$ KO vs WT (adriamycin). d) *$P<0.001$ KO vs WT (untreated), **$P<0.05$ KO vs WT (adriamycin); 6 samples per group. Experiments performed after plating cells at 20,000 and 50,000 per well demonstrated similar results.

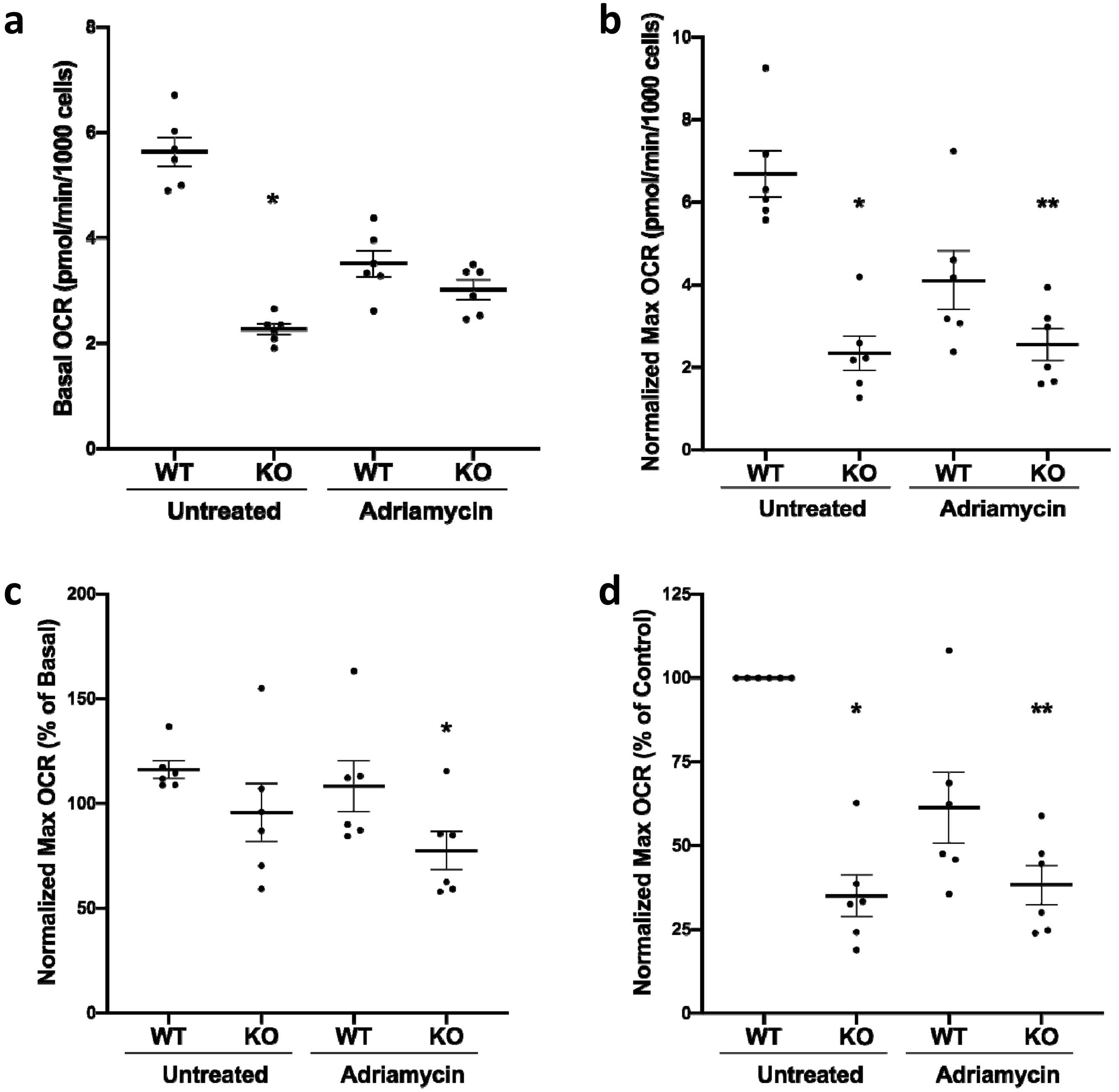

a
Basal OCR (pmol/min/1000 cells)
0
2
4
6
8
*
WT
KO
WT
KO
Untreated
Adriamycin
b
Normalized Max OCR (pmol/min/1000 cells)
0
2
4
6
8
10
*
**
WT
KO
WT
KO
Untreated
Adriamycin
c
Normalized Max OCR (% of Basal)
0
50
100
150
200
*
WT
KO
WT
KO
Untreated
Adriamycin
d
Normalized Max OCR (% of Control)
0
25
50
75
100
125
*
**
WT
KO
WT
KO
Untreated
Adriamycin

Supplementary Figure 5. Uncropped immunoblots for all figures.

Figure 2c

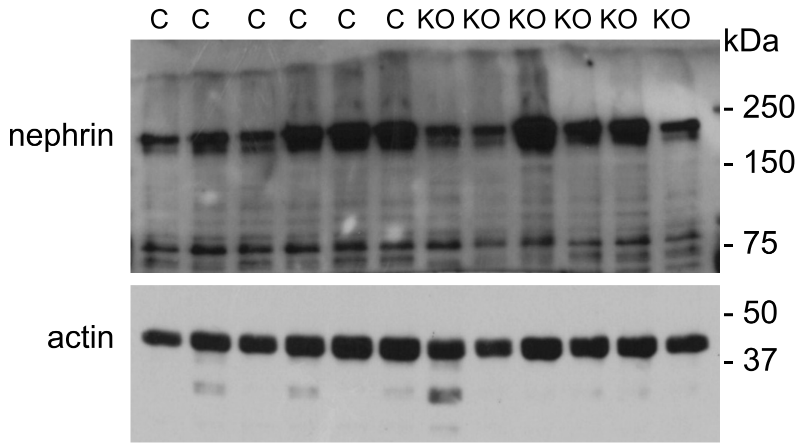

C, control/adriamycin
KO, knockout/adriamycin

Figure 3a

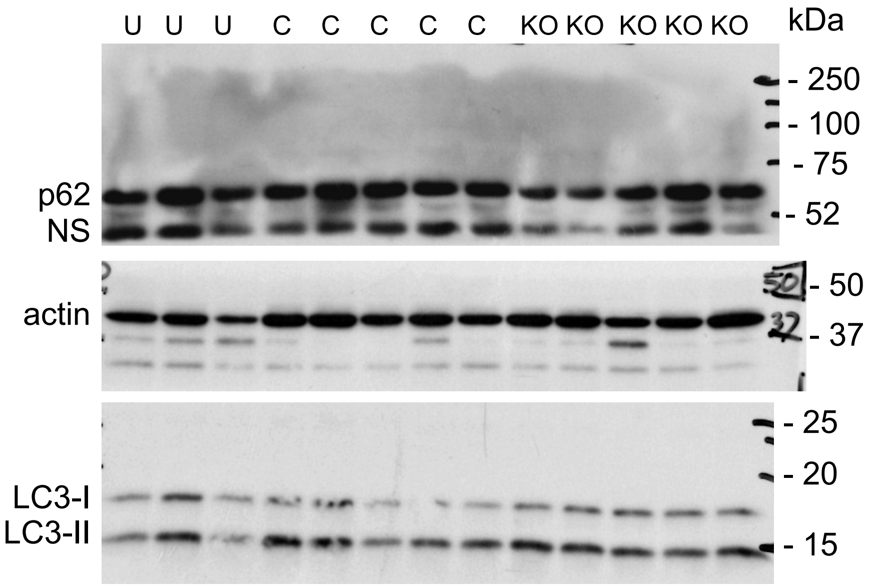

U, uninjected
C, control/adriamycin
KO, knockout/adriamycin
NS, nonspecific

Supplementary Figure 5, continued

Figure 3c

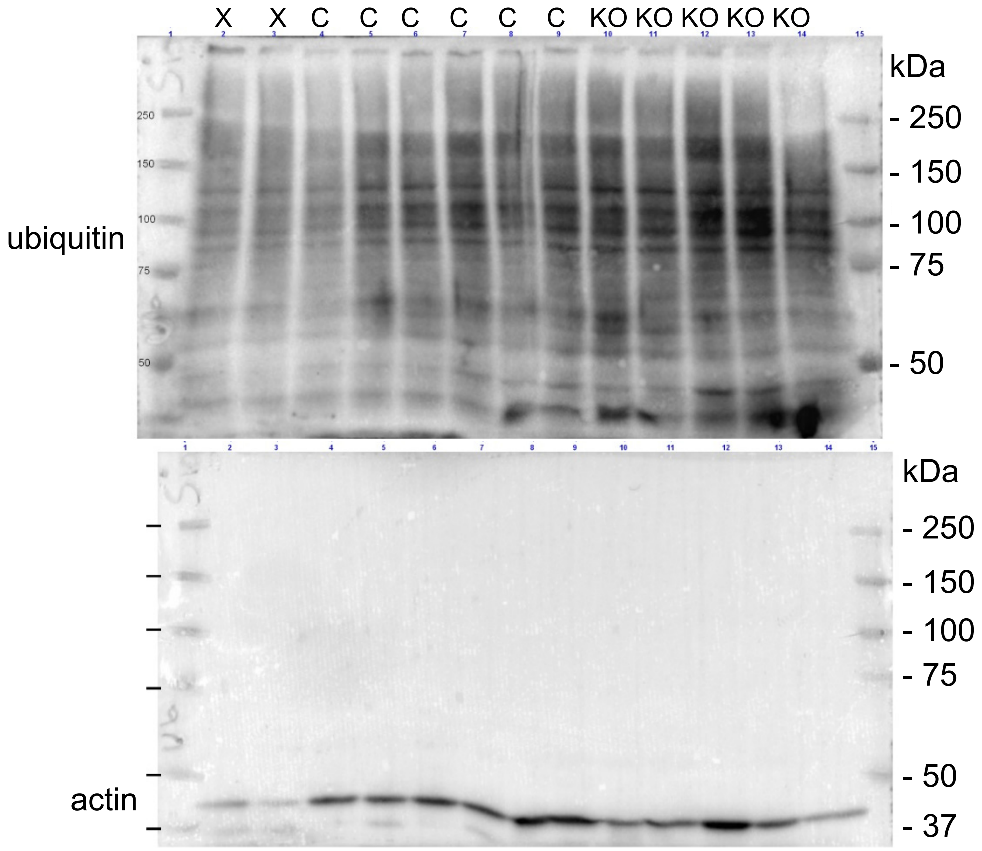

C, control/adriamycin
KO, knockout/adriamycin
X, other sample

Figure 3e

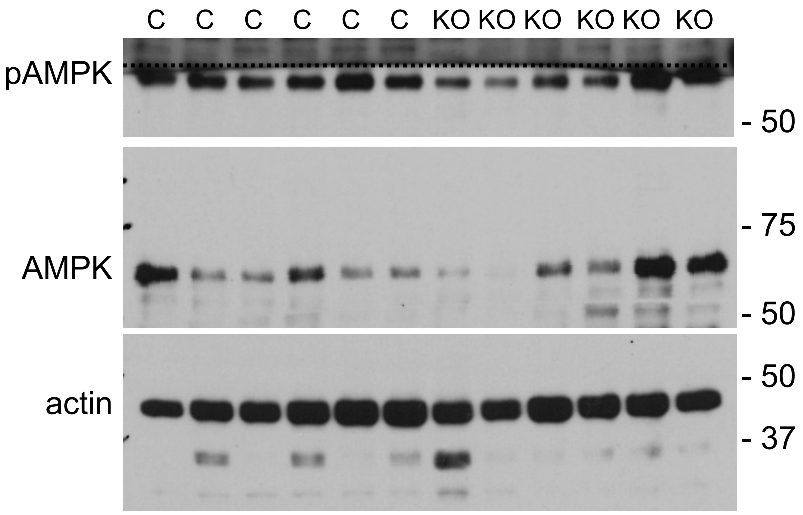

C, control/adriamycin
KO, knockout/adriamycin

Supplementary Figure 5, continued

Figure 4a

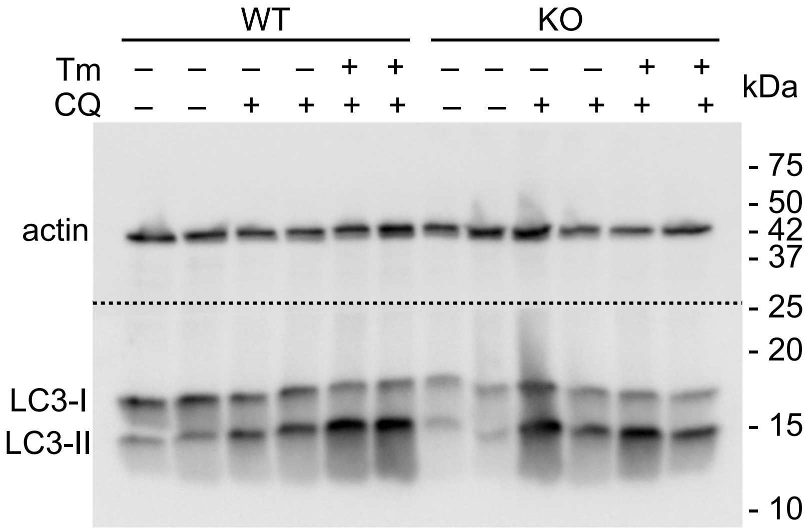

Figure 4c

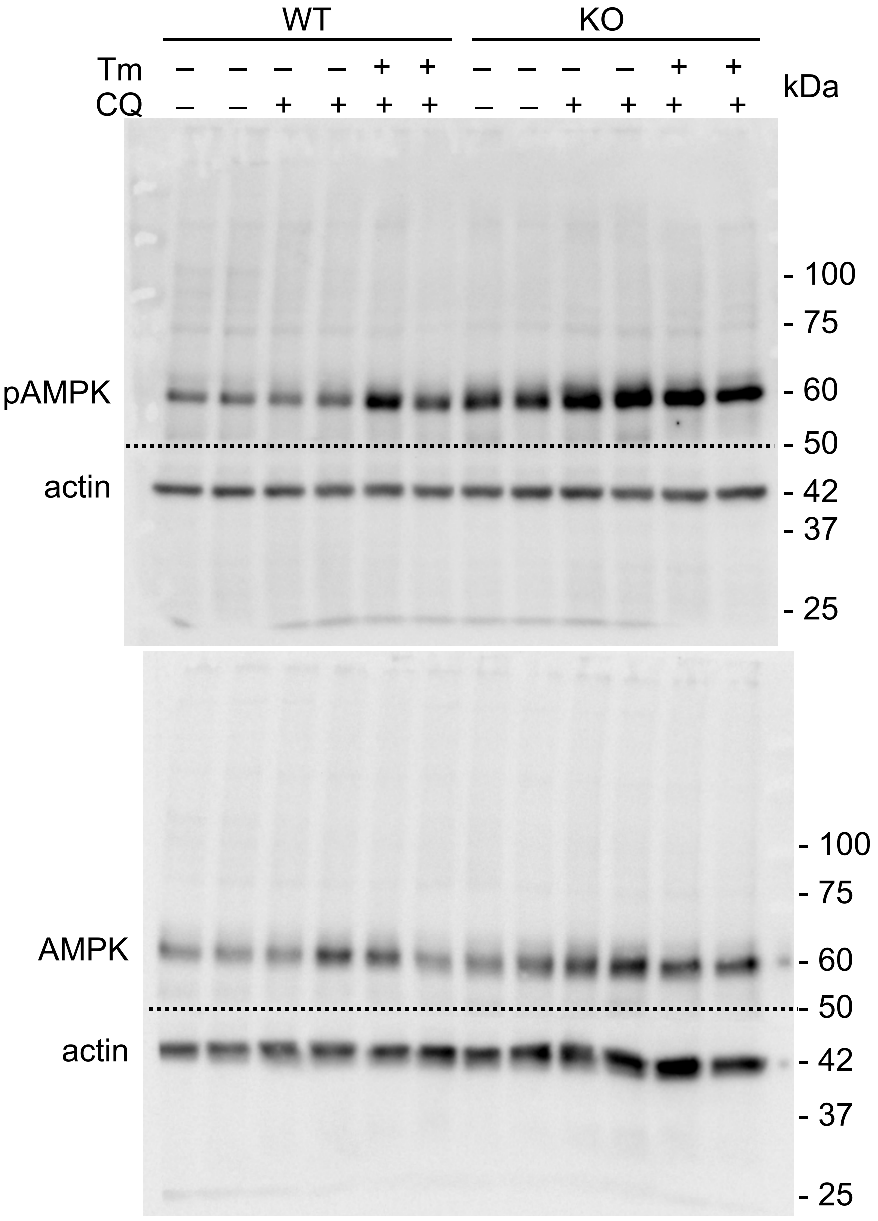

Supplementary Figure 5, continued

Figure 6a

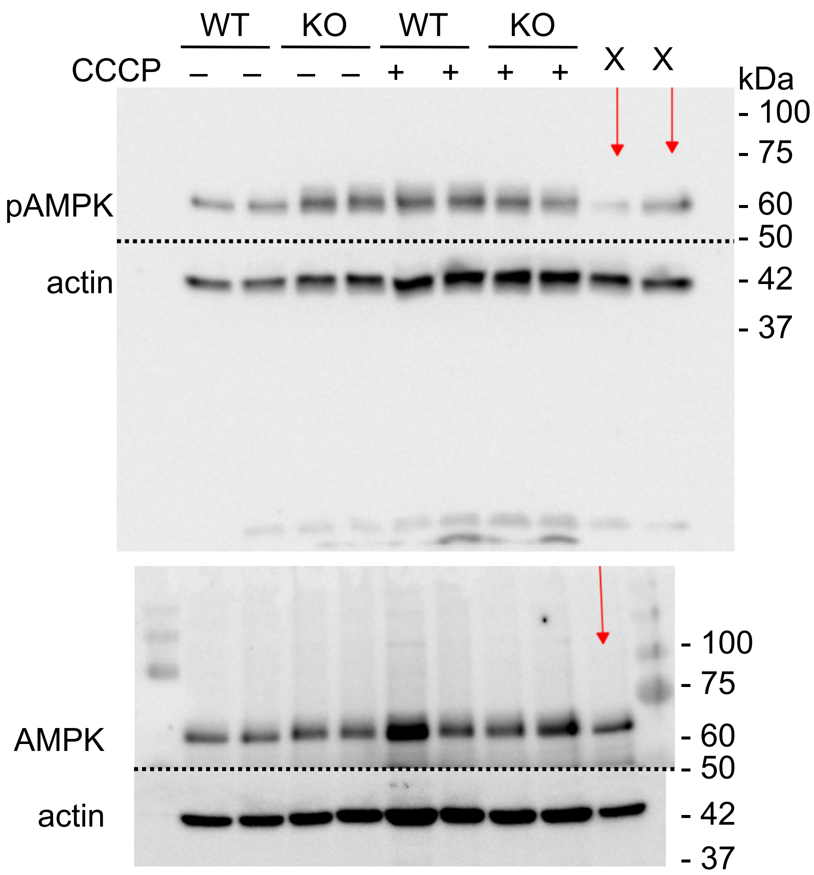

X, other sample

Figure 6c

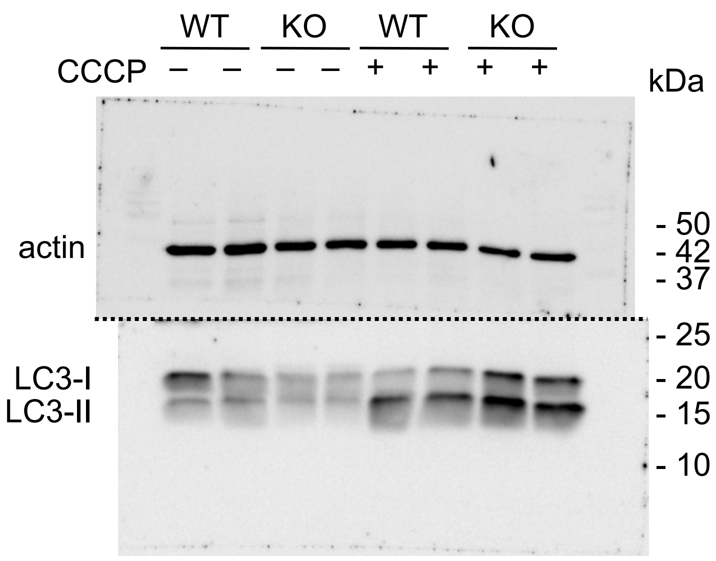

Supplement: Supplementary file 1 — Supplementary Information [file 41598_2019_52834_MOESM1_ESM.pdf]
